# Managing Conflicts of Interest in the UK National Institute for Health and Care Excellence (NICE) Clinical Guidelines Programme: Qualitative Study

**DOI:** 10.1371/journal.pone.0122313

**Published:** 2015-03-26

**Authors:** Tanya Graham, Phil Alderson, Tim Stokes

**Affiliations:** 1 King's College London, Department of Postgraduate Research, Florence Nightingale School of Nursing & Midwifery, 3.35 James Clerk Maxwell Building, 57 Waterloo Road, London SE1 8WA, United Kingdom; 2 Centre for Clinical Practice, National Institute for Health and Care Excellence (NICE), Level 1A, City Tower, Piccadilly Plaza, Manchester M1 4BT, United Kingdom; 3 School of Health and Population Sciences, College of Medical and Dental Sciences, University of Birmingham, Edgbaston, Birmingham B15 2TT, United Kingdom; Medical University of Vienna, AUSTRIA

## Abstract

**Background:**

There is international concern that conflicts of interest (COI) may bias clinical guideline development and render it untrustworthy. Guideline COI policies exist with the aim of reducing this bias but it is not known how such policies are interpreted and used by guideline producing organisations. This study sought to determine how conflicts of interest (COIs) are disclosed and managed by a national clinical guideline developer (NICE: the UK National Institute for Health and Care Excellence).

**Methods:**

Qualitative study using semi-structured telephone interviews with 14 key informants: 8 senior staff of NICE’s guideline development centres and 6 chairs of guideline development groups (GDGs). We conducted a thematic analysis.

**Results:**

Participants regard the NICE COI policy as comprehensive leading to transparent and independent guidance. The application of the NICE COI policy is, however, not straightforward and clarity could be improved. Disclosure of COI relies on self reporting and guideline developers have to take “on trust” the information they receive, certain types of COI (non-financial) are difficult to categorise and manage and disclosed COI can impact on the ability to recruit clinical experts to GDGs. Participants considered it both disruptive and stressful to exclude members from GDG meetings when required by the COI policy. Nonetheless the impact of this disruption can be minimised with good group chairing skills.

**Conclusions:**

We consider that the successful implementation of a COI policy in clinical guideline development requires clear policies and procedures, appropriate training of GDG chairs and an evaluation of how the policy is used in practice.

## Introduction

Clinical guidelines are “recommendations intended to optimize patient care that are informed by a systematic review of evidence and an assessment of the benefits and harms of alternative care options.”[1] They are seen as one of the key foundations for quality improvement in health care[2] and it is thus crucial that guidelines are of high quality. International consensus is that guidelines should be developed using an explicit and transparent process that minimizes distortions, biases, and conflicts of interest; should base recommendations on a systematic review of the existing evidence; should include experts and patient representatives on a multidisciplinary guideline development group (GDG); and should consider important patient subgroups and patient preferences.[1,3–6]

Conflicts of interest (COI) are defined as a “set of conditions in which professional judgement concerning a primary interest (such as a patient’s welfare or validity of research) tends to be unduly influenced by a secondary interest (such as financial gain).”[7] COI are an important potential source of bias in guideline development and there is increasing interest in how COI, in particular those related to the pharmaceutical industry, may adversely affect the quality of clinical guidelines.[8–10] Recent cross sectional studies of guideline group members in North America[9] and Europe (Denmark)[11] have found that financial conflicts of interest are common but that there is under-reporting of these COI by the guideline group members. There is also evidence that such COI can directly influence the development of clinical guideline recommendations.[12]

One important way of reducing COI bias in clinical guideline development is to develop and implement policies that address both the disclosure of COI by guideline group members and give clear guidance on how such COI should be handled during guideline development.[1,4] Such policies are now in general use[13] and the UK’s National Institute for Health and Care Excellence (NICE), a leading international developer of evidence based clinical guidelines,[6,14] has had a COI policy since 2004.[15,16] The NICE COI policy offers detailed guidance on what constitutes a COI, what should be disclosed and the actions that need to be taken as a result of the COI at recruitment to the guideline group and during a clinical guideline development group meeting (Table 1). In contrast to the widespread publication of COI policies[13] there has, however, been limited published research that explores in detail how such policies are actually interpreted and used by guideline producing organisations.[17] We therefore sought to determine how conflicts of interest (COIs) are disclosed and managed by a national clinical guideline developer (NICE) using qualitative methods.

**Table 1:** Summary of NICE COI Code of Practice as it relates to clinical guidelines.

Definition of types of COI
**personal pecuniary interest** involves a current (within the last 12 months) personal payment, which may either relate to the manufacturer or owner of a product or service being evaluated, in which case it is regarded as ‘**specific**’ or to the industry or sector from which the product or service comes, in which case it is regarded as ‘**non-specific**’.
**A non-personal pecuniary interest** involves payment or other benefit that benefits a department or organisation for which an individual has managerial responsibility, but which is not received personally. This may either relate to the product or service being evaluated, in which case it is regarded as ‘**specific**,’ or to the manufacturer or owner of the product or service, but is unrelated to the matter under consideration, in which case it is regarded as ‘**non-specific**’.
**A personal non-pecuniary interest** in a topic under consideration might include, but is not limited to: i) a clear opinion, reached as the conclusion of a research project, about the clinical and/or cost effectiveness of an intervention under review; ii) a public statement in which an individual covered by this Code has expressed a clear opinion about the matter under consideration, which could reasonably be interpreted as prejudicial to an objective interpretation of the evidence; iii) holding office in a professional organisation or advocacy group with a direct interest in the matter under consideration; iv) other reputational risks in relation to an intervention under review.
**A personal family interest** relates to the personal interests of a family member and involves a current payment to the family member of the employee or member. The interest may relate to the manufacturer or owner of a product or service being evaluated, in which case it is regarded as ‘**specific**’, or to the industry or sector from which the product or service comes, in which case it is regarded as ‘**non-specific**’.
**Declaration of COI**
The chair and members of the guideline development group (GDG) need to declare any COI on appointment to the GDG, annually and at each guideline development group meeting.
**Action to be taken in response to COI:**
***At appointment to GDG***
The chair the GDG must divest him/herself from any personal pecuniary interest on appointment, or as soon as practicable afterwards.
***At GDG meetings***
**Personal specific pecuniary interest**: Declare and withdraw.
**Personal non-specific pecuniary interest**: Declare and participate (unless, exceptionally, the chair rules otherwise).
**Personal family specific interest**: Declare and withdraw.
**Personal family non-specific interest**: Declare and participate (unless, exceptionally, the chair of the advisory body rules otherwise).
**Non-personal specific pecuniary interest**: Declare and participate, unless the individual has personal knowledge of the intervention or matter either through his or her own work, or through direct supervision of other people’s work. In either of these cases he or she should declare this interest and not take part in the proceedings except to answer questions.
**Non-personal non-specific pecuniary interest**: Declare and participate (unless, exceptionally, the chair of the advisory body rules otherwise).
**Personal specific pecuniary interest**: Declare and withdraw.
**Personal non-specific pecuniary interest**: Declare and participate (unless, exceptionally, the chair rules otherwise).
**Personal specific non-pecuniary interest**: Declare—action is at discretion of the chair.

## Methods

### Participants

Fifteen key informants involved in clinical guideline development at NICE: National Collaborating Centre (NCC) senior staff and GDG chairs of NICE clinical guidelines were invited to participate in the study by email. The NCCs are independent contractors whom NICE commissions to develop clinical guidelines on its behalf. GDGs chairs are independent health care professionals or lay people who are recruited through an open selection process. Access was facilitated through two of the authors (TS/PA) being known to participants as a NICE clinical guideline developer (TS) and methodologist (PA). Sampling was purposive with the aim of recruiting senior staff of NICE’s 5 NCCs from all major clinical areas including: acute care, chronic long term conditions, cancer, mental health and women’s and children’s health. We also recruited GDG chairs of guidelines published within the previous 2–3 years as these would have followed the current NICE code of practice for declaring and dealing with conflicts of interest.[16] All 15 informants agreed to take part in a confidential telephone interview conducted by an independent, experienced qualitative researcher (TG). One participant however later cancelled their involvement due to work commitments.

### Data collection

Semi- structured telephone interviews were undertaken with the 14 key informants between April and June 2012: National Collaborating Centre (NCC) senior staff (6) and GDG chairs of NICE clinical guidelines (8). The researcher (TG) explained she was external to NICE and had no particular goals in conducting the research apart from an interest in the field of quality improvement research. The interview topic guide covered the key steps of the NICE COI code of practice in terms of how it was implemented in specific clinical guidelines including: declaring COI, the ability to differentiate between different definitions of COI, taking action once COI is declared, issues and experiences whilst implementing the policy and strengths of the policy and how the policy could be improved (S1 Interview Topic Guide).

). Each interview was audio-taped and lasted between 30–60 minutes. The researcher (TG) took notes during the telephone interviews to record participants’ responses in detail. Once each interview was completed TG listened to the tape recording to check the accuracy of participant’s responses and ensured the documented responses were comprehensive account of the interview.[18]

### Data analysis

An inductive thematic analysis using the framework analysis method [19,20] was conducted on the recorded responses. TG, in consultation with TS, developed a thematic index by which the data were classified from the study aims and topic guide questions. The data were summarized and assigned to thematic charts using Excel spreadsheets. Each theme was further refined with reference to the original recorded responses. Similar themes were grouped together and relationships between themes explored. After eleven interviews similar themes were found amongst the subsequent transcripts. The recorded responses and charts were reviewed by TS and PA.

Research Ethics Committee approval in the UK was not required as the work was carried out as part of an audit of NICE’s COI policy. Verbal consent was obtained and documented from participants at the start of each interview for use in a report to NICE’s guidelines programme. Written consent from participants for the use of anonymized extracts from the interview in subsequent publications was subsequently obtained. The study authors had no access to any personal health or identifying information and all data was anonymized prior to analysis.

Data will not be deposited in a publically available resource, but the study will comply with NICE’s archiving policies.

## Results

Overall, participants reported that NICE is a well respected national organisation and the methodologies adopted by NICE are highly regarded internationally. All participants listed a number of strengths of the NICE COI policy. Strengths included being independent, open and transparent, essential for probity, and comprehensive:

*If [NICE COI policy is] implemented properly there can be no accusations of bias and no opportunity to influence. The public know that guidance is independent*.

*[Senior Staff NCC*, *Interview (I) 1]*.

*It [NICE COI policy] really does embrace all potential conflicts of interest in a way I have not seen anywhere else*.

*[NICE guideline chair*, *I6]*.

Both senior staff and chairs noted that the clarity of the policy could be improved including the use of more accessible English, providing examples and direct questioning to facilitate understanding of the policy:

*I think what was helpful was when someone who was used to applying them gave examples and described them in more approachable language*.

*[NICE Guideline Chair*, *12]*.

*You need a direct question because most forms come back with ‘none’ written on them—but I know that very few clinical people have none to declare so I will often press them on that*

*[Senior Staff NCC*, *5]*.

Participants’ responses about the process of handling COI in clinical guideline development led to two main themes: identifying and managing COIs.

### Identifying conflicts of interest

#### Self reporting

Both chairs and NCC senior staff talked about medical practitioners being unaware that their activities constituted a COI:

- *If you give a talk at [a specialist medical society] you have to put up a slide with your COI*. *When I put a slide of my conflicts—others are amazed and nobody else has any declarations of interest*. . . *most speakers had a conflict but didn’t recognise that they had one*. *[NICE guideline chair*, *I12]*

They also talked of clinical experts not perceiving themselves as being conflicted because they did consultancy work for a wide variety of companies:

- *We had an applicant that had funding from many different sources*. *In their view because they have many different interests—they did not see that as a conflict but we took a different view [Senior Staff NCC*, *I3]*

Several chairs highlighted the fact that the process of applying for and being appointed chair of a NICE committee made them realise how important COI were in the context of developing clinical guidelines:

- *Only when I was interviewed [for the chair position] did I begin to realise how wide an issue it was and how important it was to acknowledge it and recognise it rather than see it as a bar*. *[NICE guideline chair*, *I12]*

#### What constitutes a COI

Both chairs and NCC senior staff considered there was a difference between pecuniary (financial) and non-pecuniary (non-financial) COIs. Pecuniary interests were considered relatively easy to identify (e.g., share holding or paid pharmaceutical advisory board meetings). However, non-pecuniary COIs and in particular research activities were seen as both widespread and also difficult to assess in terms whether or not they constituted a COI:

- *People coming to the groups have a point of view that relates to a professional group or research you are involved in*. *You could say you are influenced by all sorts of things and so where should the line be drawn—because you are associated with a body*? *[NICE guideline chair*, *I13]*
- *Non- pecuniary personal [interests] are most difficult because it is about anything you have been outspoken about—if they publish as most academics do or do research about it you will have been outspoken about a particular treatment*. *[Senior Staff NCC*, *I3]*

Given the difficulty in making judgements on what constituted a COI one strategy used was to seek advice from other members of the guideline development centre or NICE as to whether the categorisation of a COI was correct:

*Categorisation is very subjective so I often ask for another opinion*

*[Senior Staff NCC*, *I2]*

### Managing conflicts of interest

In the NICE clinical guideline development process COI declarations need to be made at the time of recruitment of the chair and members of the GDG and also at each GDG meeting. The impact of categorising contact with the pharmaceutical industry as a COI was noted by NCC senior staff and chairs. NCC senior staff acknowledged that most experts are likely to have COI, which made decisions about appointments to GDGs complicated. Some of the chairs noted that they had made a decision to decline offers of work with industry for the period of their time as chairs in order to remain un-conflicted:

*I declared what I had done and said I would resign from my other commitments … I stopped doing consultancy work and was prepared to give this up*.

*[NICE guideline chair*, *I10]*

#### Disclosure of conflicts of interest

Both NCC senior staff and chairs talked about the fact that, as noted earlier, the process relies on self reporting of members and noted that they had to take “on trust” the information they received:

- *You are relying on them being honest and declaring what is relevant [Senior Staff NCC*, *I1]*

It was considered that non disclosure was generally the result of members not being aware as to what constituted a COI although one instance was given of a member knowingly not disclosing the full extent of his/her COI. The strategy NCC senior staff and chairs stated they used to deal with disclosure was one of repeatedly emphasising the policy at recruitment of members to the guideline and at each GDG meeting and probing clinical members if they had “nothing to declare”:

- *most [conflict of interest] forms come back with ‘none’ written on them—but I know that very few clinical people have none to declare so I will often press them on that*. *It is an issue*, *you just have to continually remind them about the policy*. . . . *There is a limit to how much we can do if they do not want to disclose*. *[Senior Staff NCC*, *I3]*
- *you can only labour the point and hope they do declare because you do not know what they get up to*. *[Senior Staff NCC*, *I1]*

#### Handling conflicts of interest at recruitment and at GDG meetings

Both chairs and NCC senior staff noted that the NICE COI policy restricted the pool of well qualified candidates for GDG positions. The policy meant that recruitment was a particular issue for chair appointments (Table 1), but this could be mitigated if the individual agreed to be appointed as a group member:

- *we do not shortlist people [for chair] we think will be conflicted—it leaves you in a difficult position as they are often leaders in the field*. . . *the people who know the most about it have the most conflicts [Senior Staff NCC*, *I5]*
- *So we asked them [chair applicant] to be a group member and not a chair so that we could ask them to leave the room*, *less problematic if they are a member than a chair*. *[Senior Staff NCC*, *I3]*.

It was also highlighted that in certain clinical areas with a lot of new therapeutic agents available clinical experts generally have COI and that this may make recruitment to GDGs difficult:

- *The ones we struggle with—there are a lot of new [disease area specified] drugs—most people are involved in the area—so we work out on a scale what is a serious conflict and what we can work with*. *It is more of a problem with recruitment [Senior Staff NCC*, *I5]*

In terms of handling COI in GDG meetings chairs and NCC senior staff emphasised that it was important to manage the group process carefully and required good chairing skills. This included being clear from the outset with GDG members about what COI categories were “problematic” and chairing the meeting in such a way to facilitate openness between members:

- *Making it clear what evidence is going to be discussed and creating an environment where people can be open [Senior Staff NCC*, *I2]*
- *We encourage all GDG members to itemise if you like all of their connections—that might be being a PI [Principal Investigator] for a drug-sponsored trial—which may mean they get statistics help for all their clinical research [NICE guideline chair*, *I14]*

The requirement to exclude members from the GDG meeting when a defined COI was discussed was, however, considered to be a breach of small group processes and as such was categorised as being a disruptive event. The disruption was described as being a source of stress for both chairs and members:

- *There was a guideline when the chair felt vulnerable—she would have valued the comments of two experts but for one question they both had a conflict [and had to leave the room]—that was difficult [Senior Staff NCC*, *I4]*
- *I had to leave the room when something was discussed that I had co-authored a paper on*, *even though I had no financial interest*. How did you feel about that? *I was a bit annoyed about that [NICE guideline chair*, *I10]*

Requiring members to leave the room was also described as having adverse effects on the task of each meeting, which was to review the relevant evidence and draft guideline recommendations. This was seen as a particular problem with clinical guideline development, where the clinical pathway for a condition and attendant multiple interventions are being considered:

- *In guideline development things are linked*. . . *someone could have a conflict for different bits of the chain*. . . *but you are discussing a whole pathway—people having to leave the room—can be very disruptive*. *[Senior Staff NCC*, *I4]*

Nonetheless several interviewees considered that the effect of this disruption could be minimised with careful group chairing:

- *Nobody loses face—sensitivities were well managed [Senior Staff NCC*, *I2]*

Another strategy that was used was to schedule the meeting agenda and dates so that those who were conflicted did not need to attend that particular session of the GDG.

## Discussion

This study offers a description of the process by which COIs are disclosed and managed by one leading international developer of clinical guidelines (NICE). Participants considered that the NICE COI policy was comprehensive and essential for probity but that the application of the policy—specifically identifying and managing COI in clinical guideline development—was not straightforward. Disclosure of COI relies on self reporting and guideline developers have to take “on trust” the information they receive. Certain types of COI (in particular, non-pecuniary) are difficult to categorise and manage and disclosed COI can impact on the ability to recruit clinical experts to GDGs.

This study specifically explores how COIs are disclosed and managed by a national guideline producing organisation. The strength of this paper is that it uses qualitative methods to explore in detail how COI policies are interpreted and used by guideline producing organisations and their GDGs. We interviewed key informants (NCC senior staff and GDG chairs) who were responsible for implementing the policy and purposively sampled to cover all major clinical areas. In terms of study limitations we were not able, due to limited time and funding, to supplement our interviews with ethnographic observation of GDG meetings which would have both provided further insights into how GDGs handle DOI and allowed us to triangulate our interview findings. It is also unclear to what extent these findings are generalisable to other national guideline producing organisations.

The findings of this study are consistent with a qualitative study exploring how research ethics committees identify and manage COI.[21] This study of US Institutional Review Boards (IRB) found that definitions of COI, in particular non-pecuniary COI, were found to be “blurry” and that un-conflicted clinical experts can be difficult to recruit. In addition, the process of handling COI in IRB meetings was seen as problematic and variable, with an “ad hoc” case by case approach used by chairs whereby members both excused themselves and were excluded by others from meetings and decision making.[21] Our study adds new knowledge to this issue of exclusion. We found it was considered both disruptive and stressful to exclude members from GDG meetings when required by the COI policy, however, the impact of this disruption can be minimised through promoting a culture of openness about interests through good chairing skills and having early and consistent discussions about COIs at GDG meetings. This finding is consistent with the principles of group dynamics which inform the recruitment and functioning of GDGs.[3]

Three findings of this study are of major importance to clinical guideline developers: self disclosure of COI, recruitment of clinical experts to GDGs and how conflicted GDG members are dealt with at GDG meetings. The NICE COI policy, in line with other national guideline producers,[13] relies on self disclosure of potential COI. It is expected that, as health care practitioners working to high ethical standards, they can be trusted to disclose any potential COI which can then be scrutinised by the chair of the relevant GDG and, as appropriate by NICE or its NCCs. It was considered by participants that non disclosure was generally the result of ignorance about the importance of COI and how they may bias decision making. The strategy used to handle this was to make it clear to GDG members from the outset what they should disclose and for COI to be asked about and noted at each meeting of the GDG. Evidence that health care professionals underreport COI in clinical guideline development[22] raises the question as to whether, as is proposed in the US,[23], [24] there should be a publically available register of COI to allow independent cross checking of disclosures. There has been recent debate whether, given that clinical experts are often conflicted, the methods of guideline development should be changed so that non clinical methodologists have primary responsibility for the clinical guideline recommendations.[8,17,25] A recent qualitative evaluation of this approach found that such a policy may be problematic to implement as clinical experts on guideline groups may not agree that non clinical methodologists should take on this lead role.[17] Although we did not formally address this issue in our study, our findings suggest that clinical experts can be involved in developing guidance that is independent, open and transparent if an appropriate COI policy is in place. We have shown that within NICE this was done primarily through not recruiting conflicted individuals as chairs of GDGs and ensuring that members with conflicts are excluded from the meeting when appropriate (Table 1).

A key recommendation from this study for guideline developers is that it is necessary but not sufficient for there to exist an explicit COI policy.[13] The implementation of a guideline COI policy often requires difficult and complex judgements to be made by senior clinicians and managers. We recommend that appropriate training of GDG chairs and members is provided to equip them with the knowledge and skills to manage reporting of COI. We would also propose that an evaluation of how the policy is used in practice is conducted to enable senior staff, chairs and members to reflect on the process of implementing the policy and offer an opportunity for improvements to be made. Such an evaluation would include triangulation of non-participant observation data from guideline development meetings with focus group and interview data to better understand how group dynamics such as disruptions can be best managed. Further research is also required to understand how non-pecuniary conflicts, such as being involved in research, impacts upon guideline development. This can guide developers in involving topic experts whilst ensuring conflicts are well managed. We consider attention to training and support for guideline developers and further evaluation is required to develop clinical guidelines we can trust.[1]

## Supporting Information

S1 Interview Topic Guide (DOCX)
