# Supplementary material for: Managing Conflicts of Interest in the UK National Institute for Health and Care Excellence (NICE) Clinical Guidelines Programme: Qualitative Study
Source: PLoS One. 2015 Mar 26;10(3):e0122313. doi: 10.1371/journal.pone.0122313 (PMC4374927; doi:10.1371/journal.pone.0122313)
Supplement: S1 Interview Topic Guide — (DOCX) [file pone.0122313.s001.docx]

**Interview topic guide:**

The interview topic guide covered the key steps of the NICE COI code of practice in terms of how it was implemented in specific clinical guidelines. The interview topic guide is presented below. The questions were the same for NCC Senior Staff and GDG Chairs. However, the prompts on occasion varied to reflect the differing roles of these individuals:

***Introductory statement***

With reference to specific NICE clinical guidelines, we are interested in your views about how NICE declaration of conflicts of interest code of practice was used and what issues were encountered.

***Question***

Is it clear when a conflict of interest (COI) should be declared?

***Prompts:***

Are the definitions of different COI clear?

• How easy is it to distinguish between personal pecuniary and non-pecuniary interests clear?

• Is it relatively easy to differentiate between specific and non-specific personal pecuniary interest?

• Is the distinction between personal and non-personal interests clear?

***Question***

Is it clear who COI should be declared to?

Is it clear what action should be taken when COI is declared?

***Prompts:***

Have there been any occasions when taking action was problematic?

• If so, tell me more about this?

Could you describe occasions when taking action was not problematic?

• What helped make taking action ‘straight forward’?

***Question***

Are there any issues implementing the policy?

***Prompts:***

For NCC Senior Staff: Have you experienced any issues regarding the recruitment of GDG chairs?

For GDG chair: did you experience any issues (yourself) regarding (your) COI when you went through the appointment process?

In your opinion do other experts think their interests or activities with industry render them non-suitable for working with NICE?

Have you experienced any issues whilst running GDG meetings (declaring interests and handling conflicts)?

Have you encountered any issues in the preparation and publication of the final guideline?

***Question***

What are the strengths of the policy?

How could the policy be improved?

Prompts:

In what ways might the explanation of the policy be changed?

How could the process be changed for the better?
